# Hemisphere Representation of Early and Later Acquired Words: Visual Identification and Lexical-Decision Tasks

**DOI:** 10.1007/s10936-025-10179-9

**Published:** 2025-12-02

**Authors:** Julio González-Alvarez, Rosa Sos-Peña

**Affiliations:** 1 Department of Basic and Clinical Psychology and Psychobiology, University Jaume I., Castellón, Spain; 2 Department of Basic, Clinical, and Biological Psychology, University Jaume I. College Health Sciences, 12071 Castellón de la Plana, Spain

**Keywords:** Age of acquisition, Written words, Hemispheric differences, Visual hemifield, Lexical decision task, Word identification

## Abstract

The relationship between the age of acquisition (AoA) of words and their cerebral hemispheric representation is controversial because the experimental results have been contradictory. While a large number of studies have found that words acquired early in life are also lateralized (as are later words) in the left hemisphere (LH), some evidence (particularly Bowers et al., 2013) suggests that early words have a bilateral (or rightward) representation because the corpus callosum is not fully developed in the early years and the two hemispheres function in parallel. In the present study, we conducted two experiments based on a divided visual hemifield paradigm, testing words that were classified according to an objective criterion of AoA. The results of both experiments were consistent and demonstrated that early words (as well as later words) are processed more quickly and efficiently in the LH, with no AoA x Hemisphere interaction. These results do not support the hypothesis of Bowers et al. (2013) and clearly show that words learned during the early years of life are also processed preferentially in the LH, similar to words learned later. These results are discussed from a theoretical standpoint, considering that all previous experiments on AoA and brain hemispheres have been conducted with adult participants.

## Introduction

The age of acquisition (AoA) of words, or *when* they are learned in life, is considered an important factor in language processing. Research shows that words acquired earlier in life tend to be processed more quickly and accurately compared to words acquired later. This effect (the *AoA effect*) has been consistently reported for various tasks: picture naming, word identification, word reading, or lexical decision tasks (see Johnston & Barry, 2006, for a review). On the other hand, words learned early in life are more resistant to brain damage in aphasia patients than words learned later in life (Brysbaert & Ellis, 2016). Cortese and Khanna (2007) found that age of acquisition predicted naming and lexical-decision performance above 22 other predictor variables, such as subjective and objective frequency, length, imageability, or neighborhood size.

One possible explanation for the AoA effect is that words acquired early in life are advantageous because they are represented bilaterally in the brain. Conversely, words learned later in life would be primarily processed in the dominant hemisphere of the brain responsible for language, typically the left hemisphere (LH) in most individuals. However, the relationship between the AoA of words and hemispheric differences in lexical processing is still a matter of debate in psycholinguistics.

The corpus callosum is a large bundle of nerve fibers connecting the homologous parts of the cerebral hemispheres. In an influential report, Gazzaniga (1974) noted that the corpus callosum grows rapidly during the first four years of life and that its myelination is not completed until several years later. He proposed that before the corpus callosum is fully developed and myelinated, the two cerebral hemispheres function in parallel and do not fully communicate with each other, as if they were two halves of a split brain. During that time, the two parts may operate more independently, with each hemisphere processing information in parallel without the same level of integration that occurs in a fully developed brain. This would have consequences for speech and language processing. The words acquired early in childhood would have a bilateral representation in the child’s (and adult’s) brain. In contrast, words acquired later would tend to have a more lateralized brain representation in the dominant (left) hemisphere.

The first empirical test of this hypothesis was conducted by Ellis and Young (1977) using a divided visual hemifield paradigm, in which words were tachistoscopically presented left or right of a central fixation point to be recognized by the participants. The stimuli were words acquired early versus later in life. However, the results did not confirm the hypothesis, and the expected interaction between the AoA and the visual hemifield (cerebral hemisphere) was not observed. They found a superiority of the LH (right visual hemifield) over the right hemisphere (RH) (left visual hemifield) in both kinds of words. Specifically, there was no significant difference in the degree of hemispheric asymmetry between early-acquired words and later-acquired words.

Two years later, Young and Bion (1980) obtained the same results using bilateral presentation of stimuli. In this paradigm, a different word was simultaneously presented in each visual hemifield, and the participants were required to recognize both words. The authors used two different times of exposure (30 and 150 ms), and, in both conditions, they observed an overall superiority of the LH (right visual hemifield) for both early and late acquired words, without any AoA x hemisphere interaction. Young et al. (1982) obtained similar results using the age of *reading* acquisition as an independent variable.

All these experiments employed words that had been rated retrospectively for AoA by adults. Boles et al. (1982) used stimuli selected from a corpus of words *actually observed* in the speech of very young children (under two years) studied by Katherine Nelson (Nelson, 1973). They did not observe differences in LH superiority in a divided visual field paradigm compared to later-acquired words. Again, the data analysis showed no interaction between the AoA and the hemispheres (visual hemifields). Beaton et al. (2007) also found an overall advantage to the LH using the same paradigm, and the laterality index was not different for early and later acquired words. This pattern was the same for monolingual English speakers and fluent bilingual English-Welsh speakers.

However, a later study obtained a different pattern of results. Bowers et al. (2013) noted that none of the prior studies had measured reaction times during lexical processing. Reaction time (RT) plays a crucial role in experimental psychology because it is a direct reflection of cognitive operations and provides valuable information about the speed and efficiency of ongoing processes. These authors conducted two experiments based on a divided visual hemifield paradigm and employed a lexical-decision task to take the RT as the main dependent variable. In the first experiment, they used early-acquired words corresponding to an AoA of 3–4 years old and later-acquired words corresponding to 7–8 years old, in both cases according to a normative study based on retrospective ratings made by adults (Gilhooly & Logie, 1980). Moreover, both sets of words were matched on several psycholinguistic variables. The analysis of the RTs of correct responses yielded a significant main effect of the AoA variable (superiority of early-acquired words) but not an effect for the visual hemifield. Crucially, the analysis showed an AoA x visual hemifield interaction. Unexpectedly, the participants responded faster to early-acquired words when the stimuli were presented in the left visual hemifield (RH). For late-acquired words, the responses were faster when the stimuli were presented in the right visual hemifield (LH). A second experiment with another set of stimuli replicated the results. According to the authors, their results were consistent with the idea that RH may play an important role in the first years of life.

However, a very recent work performed with spoken words by González-Alvarez and Cervera-Crespo (2023) found a pattern of results different from the results of Bowers et al. (2013) and analogous to the prior outcomes obtained by Ellis and Young (1977), Young and Bion (1980), Young et al. (1982), Boles et al. (1982), and Beaton et al. (2007).

González-Alvarez and Cervera-Crespo (2023) employed Spanish spoken words (AoA means: 2.9 vs. 9.3 years old) and matched them on several relevant psycholinguistic variables. The AoA was an *objective* parameter obtained from a pool of more than seven hundred children of different ages (Álvarez & Cuetos, 2007), rather than being derived from retrospective ratings by adults, subject to stereotypes or memory biases. In that study, the main dependent variable was the RT in an auditory lexical task, and the response accuracy was the secondary dependent variable. The data analysis revealed an overall superiority of the early words over the late words and that both kinds of words were processed more efficiently in the LH, with no AoA × Hemisphere interaction.

Nevertheless, the stimuli used by González-Alvarez and Cervera-Crespo (2023) were spoken words in the auditory domain, whereas those employed by Bowers et al. (2013) were printed words in the visual domain. This difference in stimulus modalities may be one of the reasons for the discrepancy between the results of the two studies. Stimuli used by González-Alvarez and Cervera-Crespo were classified according to an objective criterion of AoA with extremely distant means (2.88 vs. 9.28 years old) and carefully selected so that both groups of words matched on a set of ten relevant psycholinguistic variables. Consequently, it would be of great interest to study the cerebral hemisphere representation of the same stimuli in printed-word format, the same format as the stimuli of Bowers et al., with the same type of task (lexical decision in the visual hemifield paradigm) and analyzing the same parameter as the main dependent variable (RT). Thus, the present study would become a crucial test to determine if our results are: (A) consistent with the results of Bowers et al. (2013), that is, faster responses for early words presented to the left visual hemifield (RH) and for late words presented to the right visual hemifield (LH), or (B) consistent with the results of Ellis and Young (1977), Young and Bion (1980), Young et al. (1982), Boles et al. (1982), and Beaton et al. (2007), that is, both early and late words were more efficiently processed when presented to the right visual hemifield (LH). Furthermore, to increase the power of the present study, we added a preliminary experiment based on the task of identifying the same set of words presented briefly in the visual hemifields.

In this study, one might expect an overall superiority in processing early words compared to later words, as evidenced in the scientific literature (Cortese & Khanna, 2007; Ghyselinck et al., 2004; Johnston & Barry, 2006; Morrison et al., 1992; ). Additionally, one might anticipate a general superiority of the LH in language processing, as demonstrated by a long tradition of research dating back to the 19th century (e.g., Geschwind, 1972; Knecht et al., 2000; Krashen, 1972; Schnelle, 2010; Vigneau et al., 2006). These findings would not be novel. The main focus of the present study is the *hemispheric processing of* **early words**. Later words will likely exhibit LH superiority in line with most stimuli included in traditional research. However, what happens to early words acquired in the first years of an individual’s life? Are they also predominantly processed by the LH? Or, as Bowers et al. (2013) found, are early words predominantly processed by the RH (or both hemispheres equally, as per Gazzaniga’s hypothesis). This is the main question that the present study attempts to address.

## Experiment 1: Word Identification in Visual Hemifield

### Method

#### Participants

Participants were thirty-four young adults of both sexes (30 females) whose age range was 19–33 years (M = 20.29; SD = 2.65). All of them were psychology undergraduates at the University Jaume I (Spain), who voluntarily participated in exchange for course credit. Participants were right-handed native speakers of Spanish.

#### Materials

The stimuli consisted of 80 Spanish words composed of 40 early acquired words in childhood (AoA mean: 2,88 years old) and 40 later acquired words (AoA mean: 9,28 years old) (see Appendix). These words were previously used in spoken form in a recent work (González-Alvarez & Cervera-Crespo, 2023) and were selected from Álvarez and Cuetos (2007), a set of *objective* age of acquisition norms collected from a pool of 760 children using a picture naming task. Note that objective AoA is a more accurate measure than the usual norms based on retrospective ratings made by adults. Selected early and later acquired words used as stimuli had been matched on several relevant psycholinguistic variables obtained from the database EsPal (www.bcbl.eu/databases/espal/; Duchon et al., 2013): log (word frequency per million), number of letters, number of syllables, familiarity, imageability, concreteness, number of substitution neighbors, number of addition-letter neighbors, number of deletion-letter neighbors, and total number of neighbors (see Appendix of González-Alvarez & Cervera-Crespo, 2023).

#### Procedure

Following a similar procedure as Bowers et al. (2013), each trial started with a central presentation of a white fixation cross for 400 milliseconds on a black screen. After a 50 ms blank interval, the fixation cross was displayed in red for 50 milliseconds. After another 50 ms blank interval, the cross reappeared in white for 400 milliseconds. This sequence caused the appearance of a flickering fixation that drew the participant’s attention to the center of the screen. Then, the participant was presented with a word either to the left visual hemifield (processed by the RH) or to the right visual hemifield (processed by the LH). Each word was visually presented in capital letters for 150 ms immediately followed by a mask for 200 ms. Participants were instructed to write down the word with no time restriction. The words were presented in Arial font size 30 in white on a black background, centered within the left or right visual hemifield. Following the Bowers et al.’s (2013) procedure, this position corresponded to the word’s innermost letter extending to the left and right by 2.2º–3.1º from the center fixation point. Participants were instructed to be seated with their head 50 cm from the screen without moving their eyes from the center during the stimulus presentation. This prompt was reminded several times during the course of the experiment.

The experiment was administered by means of the PsyToolkit software (Stoet, 2010, 2017) during two sessions separated by at least 60 min of rest. Each word was presented to each of the two visual hemifields in separate sessions. In each session all the words were presented in random order and no word was repeated within the same session. The visual hemifields were counterbalanced across the two sessions and the order of the two sessions was balanced across the participants. In each session, each participant received a unique random ordering of trials.

#### Statistical Analyses

The data were analyzed using the Statistical Package for the Social Sciences (SPSS, version 29; IBM Corp, NY, 2022). For each participant, we obtained the average percentage of correct answers in each experimental condition (AoA and hemisphere). These data were analyzed obtaining the SDs, the standard error of the mean, and the confidence intervals [95% CI]. Then the data were submitted to an analysis of variance (ANOVA) to study the significance of the main factors and their interactions. We also calculated the values of the partial eta squared (η^2^_p_) to measure the effect size of the variables in the ANOVA model. This parameter calculates the proportion of variance explained by a given variable of the total variance. The effect size interpretations for η^2^_p_ values are: 0.01 = small, 0.06 = medium, and 0.14 = large (Cohen, 2013). We also calculated the observed power (oPw) for each effect to test if the experiment has sufficient statistical power, that is, the probability (from 0 to 1) of finding a significant effect.

### Results and Discussion

**Fig. 1:**
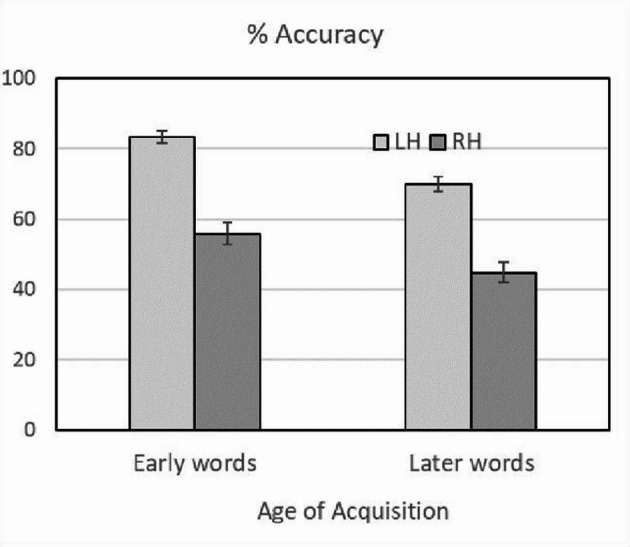
Experiment 1. Percentages of correct responses as a function of the Age of Acquisition of words for the left hemisphere (LH) and right hemisphere (RH). Error bars represent plus or minus one standard error of the mean

Accuracy to word stimuli was 63.5% overall, although varied significantly depending on the cerebral hemisphere and the age of acquisition of the words. Percentage of correct responses for early acquired words were: for the LH 83.2% (SD = 10.1%), 95% CI [79.8%, 86.6%]; for the RH: 55.8% (SD = 18.2%), [49.7%, 61.9%]. For later acquired words percentages were: for the LH 69.9% (SD = 12.3%), [65.80%, 74.1%]; for the RH 44.9% (SD = 17.0%), [39.1%, 50.6%] (see Fig. 1).

A 2 (Age of Acquisition, early vs. later words) x 2 (Hemisphere, left vs. right) ANOVA was performed on percentage of correct responses, and we made separate analyses across participants (*F*_1_) and items (*F*_2_). The analysis revealed a significant main effect of Age of Acquisition through subjects, *F*_1_(1, 33) = 135.32, MS_e_ = 36.98, *p* < .001, η^2^_p_ = 0.804, oPw = 1, and through items, *F*_2_(1, 78) = 7.08, MS_e_ = 879.09, *p* < .01, η^2^_p_ = 0.083, oPw = 0.748, because early acquired words obtained larger accuracy (mean 69.5%) than later acquired words (57.4%). Also, the analysis found a significant main effect of Hemisphere through subjects, *F*_1_(1, 33) = 132.94, MS_e_ = 176.23, *p* < .001, η^2^_p_ = 0.801, oPw = 1 and through items, *F*_2_(1, 78) = 247.72, MS_e_ = 112.56, *p* < .001, η^2^_p_ = 0.761, oPw = 1, because the LH yielded larger accuracy (mean 76.6%) than the RH (50.3%).

Importantly, the ANOVA did not reveal an Age of Acquisition x Hemisphere interaction effect neither through subjects (*F*_1_ = 1.65; *p* = .208) nor through items (*F*_2_ < 1), since the accuracy disparities between RH vs. LH were not significantly different for early acquired words (27.4%) than for later acquired words (25.1%). As previously stated, the main focus of the present study is the **hemispheric representation of early words**, so we tested whether the difference 83.2% vs. 55.8% in favor of LH was statistically significant. To this end, we conducted an ANOVA of the percentages of correct responses to early words, separated by brain hemispheres. The hemisphere effect was clearly significant through subjects, *F*_1_(1, 33) = 122.88, MS_e_ = 104.069, *p* < .001, η^2^_p_ = 0.788, oPw = 1 and through items, *F*_2_(1, 78) = 130.44, MS_e_ = 115.11, *p* < .001, η^2^_p_ = 0.770, oPw = 1.

The results of Experiment 1 are very clear: the percentages of correct responses indicated that the LH (right visual hemifield) is more efficient than the RH (left visual hemifield) in recognizing words presented very briefly in a visual hemifield. More importantly, this left-brain superiority occurs for words learned early in life and words learned later in life. These data suggest that the LH, which is dominant for language processing in the majority of individuals, maintains its processing advantage for linguistic information regardless of the timing of word acquisition. On the other hand, the results of this experiment have revealed the general superiority of early words in being recognized better than late words, despite being equal in lexical frequency and nine additional parameters.

## Experiment 2: Lexical Decision

### Method

#### Participants

Participants were seventy young adults of both sexes (58 females) whose age range was 19–35 years (M = 20.53; SD = 2.52). All of them were psychology undergraduates at the University Jaume I (Spain), who voluntarily participated in exchange for course credit. Participants were right-handed native speakers of Spanish and none of them had participated in the Experiment 1.

#### Materials

The stimuli were the same 80 Spanish words used in the Experiment 1 plus 80 additional nonwords used as fillers (see Appendix). All nonwords were pronounceable and created by changing one letter from real and common Spanish words (not included in the experimental set).

#### Procedure

It was the same as the procedure used in the Experiment 1 with the following differences. Each stimulus was also displayed for 150 ms in one of the two visual hemifields, but no mask was subsequently presented. Participants performed a lexical decision task in which they had to decide as quickly and accurately as possible whether each stimulus was a Spanish word or a nonword. They indicated their decision by pressing one of two keys on the computer keyboard (the “A” key with the left forefinger, and the “L” key with the right forefinger; for half of the participants, the “A” indicated a nonword response and the “L” indicated a word response; and for the other half these responses were reversed).

As in the Experiment 1, each stimulus was presented in both visual hemifields in different sessions in counterbalanced order. Within each session, no stimulus was repeated. The order of the two sessions was counterbalanced across the participants. In each session, each participant received a unique random ordering of trials.

#### Statistical Analyses

We applied the same statistical analysis as in Experiment 1, but now on the RTs, which is the main dependent variable of Experiment 2, and also on accuracy of responses.

### Results and Discussion

**Fig. 2:**
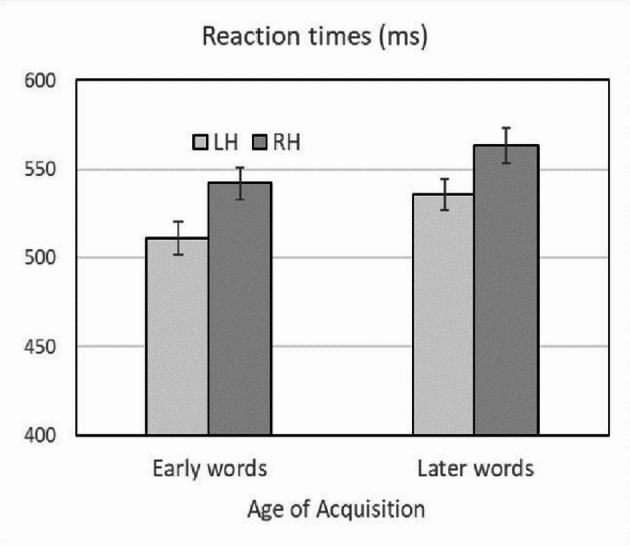
Experiment 2. Mean reaction times in milliseconds (ms) as a function of the Age of Acquisition of words for the left hemisphere (LH) and right hemisphere (RH). Error bars represent plus or minus one standard error of the mean

We considered reaction times (RTs) of correct responses to word stimuli. RTs smaller than 200 ms and larger than 1159 ms (Mean + 2.5SD) were excluded from analysis. Mean RTs for early acquired words were the following: for the LH: 511.1 ms (SD = 77.5), 95% confidence interval (CI) = [492.9, 529.3]; for the RH: 542.0 ms (SD = 78.2), 95% CI = [523.7, 560.3]. For later acquired words RTs were the following: for the LH: 535.6 ms (SD = 73.4), 95% CI = [518.4, 552.8]; for the RH: 563.2 ms (SD = 82.5), 95% CI = [543.9, 582.5] (see Fig. 2).

A 2 (AoA, early vs. later words) × 2 (hemisphere, left vs. right) ANOVA was performed on RTs, and we carried out separate analyses across participants (*F*_*1*_) and items (*F*_*2*_). The analysis obtained a significant main effect of AoA through subjects, *F*_*1*_(1, 69) = 63.56, MSe = 577.25, *p* < .001, ηp^2^ = 0.479, oPw = 1, and through items, *F*_*2*_(1, 78) = 6.04, MSe = 1843.55, *p* = .016, ηp^2^ = 0.072, oPw = 0.680, because early acquired words yielded smaller RTs (526.5 ms) than later acquired words (549.4 ms). Also, the analysis found a significant main effect of hemisphere through subjects, *F*_*1*_(1, 69) = 23.62, MSe = 2540.01, *p* < .001, ηp^2^ = 0.255, oPw = 0.998, and through items, *F*_*2*_(1, 78) = 47.24, MSe = 1049.34, *p* < .001, ηp^2^ = 0.377, oPw = 1, indicating that the LH yielded smaller RTs (mean 523.3 ms) than the RH (552.6 ms). Importantly, the analysis did not reveal an AoA × Hemisphere interaction effect neither through subjects (*F*_*1*_ < 1) nor through items (*F*_*2*_ < 1), since the RT disparities between RH versus LH were not significantly different for early acquired words (17 ms) than for later acquired words (16 ms). We performed an ANOVA with the RTs to *early words*, considering brain hemispheres as a factor, to test whether the difference of RTs (511.1 ms vs. 542.0 ms) in favor of LH (shorter RTs) was statistically significant. The results clearly showed significance through subjects *F*_*1*_(1, 69) = 21.56, MSe = 1587.452, *p* < .001, ηp^2^ = 0.234, oPw = 0.995, and through items, *F*_*2*_(1, 39) = 26.13, MSe = 509.044, *p* < .001, ηp^2^ = 0.401, oPw = 0.999.

**Fig. 3:**
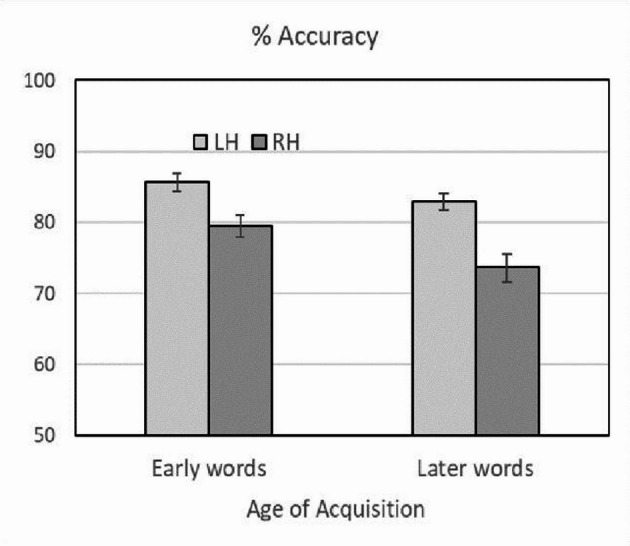
Experiment 2. Percentages of correct responses as a function of the Age of Acquisition of words for the left hemisphere (LH) and right hemisphere (RH). Error bars represent plus or minus one standard error of the mean

As a secondary dependent variable, we examined the accuracy of responses. Percentages of correct responses for early acquired words were 85.6% (SD = 10.8%), 95% CI [83.1%, 88.2%] for the LH; and 79.5% (SD = 12.6%), [76.5%, 82.4%] for the RH. Percentages of correct responses for later acquired words were 82.9% (SD = 10.1%), 95% CI [80.6%, 85.3%] for the LH; and 73.6% (SD = 16.6%), [68.8%, 77.5%] for the RH (see Fig. 3).

A 2 (AoA, early vs. later words) x 2 (Hemisphere, left vs. right) ANOVA was carried out on proportions of correct responses and we made separate analyses across participants (*F*_1_) and items (*F*_2_). The analysis found a significant main effect of Age of Acquisition through subjects, *F*_1_(1, 69) = 55.60, MS_e_ = 23.01, *p* < .001, η^2^_p_ = 0.446, oPw = 1, and through items, *F*_2_(1, 78) = 4.36, MS_e_ = 178.11, *p* = .040, η^2^_p_ = 0.053, oPw = 0.541, because early acquired words obtained larger accuracy (mean 82.6%) than later acquired words (78.3%). The analysis also found a significant main effect of hemisphere through subjects, *F*_*1*_(1, 69) = 24.99, MSe = 166.76, *p* < .001, ηp^2^ = 0.266, oPw = 0.999, and through items, *F*_*2*_(1, 78) = 80.65, MSe = 26.68, *p* < .001, ηp^2^ = 0.508, oPw = 1, because the LH obtained larger accuracy (mean 84.3%) than the RH (76.6%). The AoA × Hemisphere interaction effect was significant through subjects *F*_*1*_(1, 69) = 4.03, MSe = 42.73, *p* = .049, ηp^2^ = 0.055, oPw = 0.508, but not through items, *F*_*2*_(1, 78) = 2.52, MSe = 26.68, *p* = .117, ηp^2^ = 0.031, oPw = 0.348. We also performed an ANOVA with the proportions of correct responses to *early words*, considering brain hemispheres as a factor, to test whether the difference (86.5% vs. 79.5%) in favor of LH was statistically significant. The results clearly showed significance through subjects *F*_*1*_(1, 69) = 15.98, MSe = 82.754, *p* < .001, ηp^2^ = 0.188, oPw = 0.976, and through items, *F*_*2*_(1, 39) = 30.57, MSe = 23.846, *p* < .001, ηp^2^ = 0.439, oPw = 1.

The pattern of results in this experiment is a replica of the pattern shown in the first experiment, in this case with RTs in a lexical decision task as the main dependent variable. As expected, responses to early acquired words were faster than those to later acquired words, reflecting that the former are processed more quickly and efficiently than the latter. As expected, responses to words presented to the LH (right visual field) were overall shorter than responses to those same words presented to the RH (left visual field), reflecting the superiority of the dominant hemisphere for language processing. More importantly, for our purposes, it should be noted that LH superiority was manifested for *both* early and late words, not just for late words (Fig. 2). An examination of response accuracy as a secondary variable parallels these results and presents the same pattern (Fig. 3). In the next section, we will discuss the theoretical implications of the results of both experiments for the hypotheses presented in the introduction.

## General Discussion

The present study is framed by the question of word’s brain representation (specifically their hemispheric laterality) and the AoA of these words, comparing the processing of words learned early in the first years of life versus words learned later. Since the 1970s, several empirical investigations have found consistent results, both with visual stimuli (written words: Beaton et al., 2007; Boles et al., 1982; Ellis & Young, 1977; Young & Bion, 1980; Young et al., 1982), and, more recently, auditory stimuli (spoken words: González-Alvarez & Cervera-Crespo, 2023) indicating that early words (like late words) are predominantly processed by the LH. The only study with different results was that of Bowers et al. (2013), who expectedly found LH superiority in processing later words but an unexpected RH superiority in processing early words (shorter RTs). Unlike those of Bowers et al. (2013), our results clearly demonstrate that early-word processing is significantly superior in the LH compared to the RH, and not vice versa.

We carried out two experiments based on the visual hemifield paradigm. The first was based on identifying words presented very briefly, and the second was based on the lexical decision task. The two experiments yielded the same pattern of results. As expected, according to previous research, words acquired earlier in life were processed more quickly and efficiently than words acquired later in life, being equal in lexical frequency and nine additional relevant psycholinguistic variables. This phenomenon is often referred to as the “age of acquisition (AoA) effect,” and various theoretical reasons attempt to explain its nature, such as a richer semantic representation of early words with stronger connections with other words during a period of more neuroplasticity.

On the other hand, the results of both experiments coincide in showing the left cerebral hemisphere to be more efficient for lexical processing than the RH. It is also an expected result, as there is a long tradition of research, dating back to Broca’s reports from the 19th century, showing that in most people (especially right-handed people), the LH is dominant for language processing.

Beyond the main effects, the relationship between the variables AoA and cerebral hemispheres is crucial for the objective of the present study. More specifically, we expected an LH advantage for later words because this asymmetric effect aligns with the long-standing traditional research (mentioned above) on the general superiority of the LH in language processing. However, the question regarding the early words acquired by individuals in their first years of life still remains. The experimental evidence from the present study leaves no room for doubt; these early words are also processed more quickly and efficiently by the LH than the RH, just like words learned later. Moreover, this superiority of the LH is similar in both groups of words, without interaction between the AoA and the cerebral hemisphere. Consequently, our results do not support the hypothesis of Gazzaniga (1977). This author stated that words learned very early in childhood would have a bilateral brain representation, or at least a representation more bilateral than that of words learned later. This would be because the corpus callosum, or the anatomical structure that connects the two cerebral hemispheres, had not matured and sufficiently myelinated to connect the two brain parts effectively. Functionally, it would appear as if the child has a split brain in which both hemispheres act in parallel. On the contrary, once the corpus callosum had matured and language had specialized in the dominant (left) hemisphere, the words learned later would have a more asymmetric representation focused on the LH.

Experimental tests of this hypothesis with studies conducted on adult participants have not shown this to be true. Most experiments have found that both words acquired early in life and those acquired later have a lateralized representation in the left cerebral hemisphere to the same degree (Beaton et al., 2007; Boles et al., 1982; Ellis & Young, 1977; Young & Bion, 1980; Young et al., 1982). González-Alvarez and Cervera-Crespo (2023) pointed out that all previous studies had been conducted with stimuli consisting of written words. They reasoned that if scientists want to compare the processing of words acquired very early (before the child can read) versus words acquired later, it seems more logical to use spoken words as experimental stimuli. Consequently, these authors used spoken words and, again, obtained the overall pattern of the superiority of the LH for both early and late words.

Surprisingly, Bowers et al. (2013) found a different pattern of results. They noted that none of the prior studies had measured reaction times (RT) during the lexical processing and conducted two experiments employing a lexical-decision task with printed words and taking the RTs as a more accurate measure of the ongoing processes. Against the general trend, Bowers et al. found that early words were not only not represented bilaterally in the brain but were also processed slightly better (with shorter reaction times) in the RH. These authors explained their results by suggesting that the RH may play a relevant role in the first three years of life (Chiron et al., 1997; Dehaene-Lambertz et al., 2006).

We conducted the present study matching the experimental conditions of Bowers et al. (2013), using printed words as stimuli, and applying the same lexical decision task in a visual hemifield paradigm with RTs as the main dependent variable. Our results have been very clear (LH dominance for early words), and contradictory to those of Bowers et al. (RH dominance for early words), and in line with a large number of previous experiments. The difference between our results and those of Bowers et al. (2013) could possibly be methodological, particularly with regard to the chosen stimuli. Bowers et al. (2013, p. 185) stated, “Because the present studies tested stimuli that were exclusively highly imageable nouns, we acknowledge that the results may not generalize to other types of words”. Furthermore, Bowers et al. (2013) matched their stimuli for lexical frequency (early vs. later words) using obsolete word frequency norms (Francis & Kucera, 1982). This is a significant consideration, as lexical frequency is a robust variable that can obscure the effects of other less influential variables. In Bowers et al.’s (2013) study, half of the words were presented to the left visual hemifield (that is, RH) and the other half to the right visual hemifield (LH), meaning that each word did not serve as its own control with respect to the cerebral hemispheres. In our study, each word was presented to both visual hemifields/hemispheres (in separate sessions, in random order, and with visual hemifields counterbalanced across the sessions), allowing each word to act as its own control with respect to the hemispheres.

Another possible reason is the difference in the language of the stimuli; Bowers et al. (2013) used English words, while we used Spanish words. There are important differences between English and Spanish in several aspects. For example, the orthography of English is considered to be opaque, while that of Spanish is regarded as being much shallower (Whitley, 2002), which could have implications for word processing. For instance, in reading aloud, AoA effects have been found in Spanish with highly imageable words (Cuetos & Barbon, 2006), but not with less imageable words (Davies et al., 2014). Conversely, in English, Cortese and Schock (2013) found that AoA effects were more pronounced with less imageable words than with highly imageable words. However, the argument regarding the language of the stimuli loses strength when considering that five studies (Beaton et al., 2007; Boles et al., 1982; Ellis & Young, 1977; Young & Bion, 1980; Young et al., 1982), that yielded results contrary to those of Bowers et al. (2013) and consistent with our findings, used English words.

This pattern of results (LH dominance for early words) raises two questions (González-Alvarez & Cervera-Crespo, 2023):

1. Were the early words acquired laterally in the LH from the beginning, despite the fact that the corpus callosum was not fully mature? Indeed, there is evidence that the structural and functional asymmetry of the language network appears much earlier in childhood than we had thought (Dehaene-Lambertz et al., 2006; Reynolds et al., 2019), and that the arcuate fasciculus, which connects the Broca and Wernicke areas, is fully established by two years of age (Reynolds et al., 2019).
2. Did the early words initially have a bilateral representation—or even a rightward representation—but later, given the plasticity of the brain and with use over the years, were they lateralized toward the speech hemisphere? Particularly the orthographic and phonological representations. It is important to highlight that all the experiments conducted so far to study the relationship between the age of acquisition of words and their hemispheric processing have been performed with adult participants. Consequently, we cannot rule out this second option a priori.

Further research is needed to fully understand the developmental trajectory of these lexical processes through longitudinal studies tracing individuals from infancy to early adulthood. Combining behavioral data with neuroimaging (fMRI) or electrophysiological (ERP) measurements would allow researchers to investigate the neural correlates of word processing and determine if there are age-related changes in lateralization patterns. It could help clarify whether early acquired words are initially processed bilaterally (or in the RH) and how lateralization progresses with language development.

## Data Availability

Data can be made available upon request from the first author in a form that discloses personal information of the participants.

## Appendix

Experimental stimuli used in the two experiments.

**Table:**

Words used as stimuli in the experiments 1 and 2			
Early acquired words	Later acquired words	Nonwords used in the Experiment 2	
PEZ [fish]	RED [net]	ANGIL	MARISCE
CAMA [bed]	RANA [frog]	APIE	MARO
DEDO [finger]	CUBO [cube]	BACADOR	MECEDIRA
GATO [cat]	MURO [wall]	BAMBE	MENTO
LEON [lion]	COPA [cup]	BEDA	METELI
MESA [table]	MAGO [wizard]	BITON	MIAL
PATO [duck]	NUBE [cloud]	BOCINE	MIEN
TREN [train]	SOFA [sofa]	BORDILLE	MOSTAZO
VACA [cow]	FOTO [photo]	BRISERO	MOTAJE
VASO [glass]	POZO [well]	CAFO	MOTIL
ARBOL [tree]	AGUJA [needle]	CALABIZO	NAVAJE
AVION [airplane]	BANCO [bank]	CAON	PAELLE
BARCO [boat]	CABRA [goat]	CASCE	PALE
COCHE [car]	MOSCA [fly]	CEDERA	PASTOLA
GLOBO [balloon]	ZORRO [fox]	CERAMELO	PEN
RELOJ [clock]	GALLO [rooster]	CERTON	PERE
LLAVE [key]	TUMBA [grave]	CERVEZO	PESCINA
PEINE [hair comb]	OREJA [ear]	CHIZA	PESTA
PERRO [dog]	CISNE [swan]	CHOREZO	PILO
RUEDA [wheel]	ARAÑA [spider]	CIRROZA	PINZO
SILLA [chair]	BALON [ball]	COLIFLAR	PIÑE
CAMION [truck]	CAMISA [shirt]	CORNASA	PLUN
CONEJO [rabbit]	MEDICO [doctor]	DARDE	PORSIANA
CUERDA [string]	COCINA [kitchen]	DERDO	PULLO
ESCOBA [broom]	FUENTE [fountain]	ESCADO	RASTRELO
PAYASO [clown]	JERSEY [sweater]	ESTACI	REDIADOR
PELOTA [ball]	PATATA [potato]	FABIDA	SARDENA
ZAPATO [shoe]	ANILLO [ring]	FANTE	SARTIJA
CUCHARA [spoon]	CASCADA [waterfall]	FERO	SEL
PLATANO [banana]	VENTANA [window]	FIELLE	SELON
TENEDOR [fork]	CEBOLLA [onion]	FLEN	SELSA
CAMPANA [bell]	CHALECO [jacket]	FOLETE	SENDIRO
CARACOL [snail]	FABRICA [factory]	FOLIE	SENTERO
MANZANA [apple]	HORMIGA [ant]	FRISA	SICO
PISTOLA [gun]	CEPILLO [brush]	GALLETO	SITA
ELEFANTE [elephant]	ACORDEON [accordion]	GUISI	TARTILLA
CUCHILLO [knife]	ANTORCHA [torch]	HELIDO	TERTA
ESTRELLA [star]	PANCARTA [banner]	LENO	TINEL
MARTILLO [hammer]	LEOPARDO [leopard]	MADUJA	VENO
PARAGUAS [umbrella]	CINTURON [belt]	MANTE	YOGAR

In brackets, English translations of the words
